# Feasibility and safety of a 6-month exercise program to increase bone and muscle strength in children with juvenile idiopathic arthritis

**DOI:** 10.1186/s12969-018-0283-4

**Published:** 2018-10-22

**Authors:** Kristin M. Houghton, Heather M. Macdonald, Heather A. McKay, Jaime Guzman, Ciarán Duffy, Lori Tucker, on behalf of the LEAP Study Investigators, Roberta Berard, Gilles Boire, Alessandra Bruns, Sarah Campillo, Gaëlle Chédeville, Paul Dancey, Janet Ellsworth, Debbie Feldman, Adam Huber, Nicole Johnson, Roman Jurencak, Claire LeBlanc, Deborah Levy, Paivi Miettunen, Kimberly Morishita, Suzanne Ramsey, Alan Rosenberg, Johannes Roth, Dax Rumsey, Heinrike Schmeling, Rosie Scuccimarri, Natalie Shiff, Elizabeth Stringer, Shirley Tse, Leanne Ward

**Affiliations:** 1 Division of Rheumatology, K4-123 ACB, British Columbia Children’s Hospital, 4480 Oak Street, Vancouver, BC V6H 3V4 Canada; 2 Department of Pediatrics, University of British Columbia, Vancouver, BC Canada; 3 Department of Family Practice, University of British Columbia, Vancouver, BC Canada; 4 Centre for Hip Health and Mobility, Vancouver Coastal Health Research Institute, Vancouver, BC Canada; 5 Department of Orthopaedics, University of British Columbia, Vancouver, BC Canada; 6 Division of Rheumatology, Department of Pediatrics, Children’s Hospital of Eastern Ontario and University of Ottawa, Ottawa, Canada

**Keywords:** Exercise, Physical activity, Juvenile arthritis, Muscle, Bone

## Abstract

**Background:**

Arthritis in childhood can be associated with muscle weakness around affected joints, low bone mass and low bone strength. Exercise is recognized as an important part of management of children with juvenile idiopathic arthritis (JIA) but the exercise prescription to best promote bone and muscle health is unknown. We therefore aimed to: 1. assess feasibility and safety of a 6-month home- and group-based exercise program for children with JIA; 2. estimate the effect of program participation on bone mass and strength, muscle function and clinical outcomes and 3. determine if any positive changes in bone and muscle outcomes are maintained 6 months later.

**Methods:**

We recruited 24 children with JIA who were part of the Linking Exercise, Physical Activity and Pathophysiology in Childhood Arthritis (LEAP) study to participate in a 6-month home-based exercise program involving jumping and handgrip exercises, resistance training and one group exercise session per month. We assessed lumbar spine bone mass (dual energy X-ray absorptiometry), distal tibia and radius bone microarchitecture and strength (high-resolution peripheral quantitative computed tomography), muscle function (jumping mechanography, dynamometry) and clinical outcomes (joint assessment, function, health-related quality of life) at baseline, 6- and 12-months. Adherence was assessed using weekly activity logs.

**Results:**

Thirteen children completed the 6-month intervention. Participants reported 9 adverse events and post-exercise pain was rare (0.4%). Fatigue improved, but there were no other sustained improvements in muscle, bone or clinical outcomes. Adherence to the exercise program was low (47%) and decreased over time.

**Conclusion:**

Children with JIA safely participated in a home-based exercise program designed to enhance muscle and bone strength. Fatigue improved, which may in turn facilitate physical activity participation. Prescribed exercise posed adherence challenges and efforts are needed to address facilitators and barriers to participation in and adherence to exercise programs among children with JIA.

**Trial registration:**

Data of the children with JIA are from the LEAP study (Canadian Institutes of Health Research (CIHR; GRANT# 107535). http://www.leapjia.com/

**Electronic supplementary material:**

The online version of this article (10.1186/s12969-018-0283-4) contains supplementary material, which is available to authorized users.

## Background

Physical activity (PA) during childhood and adolescence is essential for optimal growth and development. However, compared with their healthy peers, children with juvenile idiopathic arthritis (JIA) are less physically fit, spend less time engaged in moderate to vigorous physical activity (MVPA) [1–4], and more often choose sedentary pursuits [5]. Physical inactivity may in turn exacerbate JIA symptoms and prevent children with JIA from achieving recommended levels of physical activity [6]. In keeping with an ‘exercise is medicine’ approach, PA is considered an important non-pharmacological therapy for children with JIA [7, 8].

A variety of exercise programs have been evaluated in children with JIA (reviewed in [9–11]) but it is unclear what specific exercise prescription best promotes bone and muscle health. Weight-bearing PA and muscle strengthening exercises are essential for optimal bone mass and strength accrual [12]. In children with JIA, bone accrual may be hindered by the combined effects of inflammatory cytokines, glucocorticoid therapy, low muscle mass and physical inactivity [13]. As a result, children with JIA are twice as likely to have low areal bone mineral density (aBMD by dual energy X-ray absorptiometry, DXA) as compared with their healthy peers [14, 15] as well as deficits in bone structure and strength as estimated with three-dimensional imaging tools [16, 17]. In turn, these bone deficits are associated with a 1.5–3 times higher risk of fracture among children with JIA [18]. Thus, strategies are needed to target musculoskeletal health in children with JIA. To date, only one study investigated the effects of a home-based exercise program on bone mass in children with JIA [8, 19]. However, this exercise program lasted only 12 weeks, which is likely insufficient to assess true benefits in bone accrual [20].

DXA is the current clinical gold standard for monitoring bone health; however, a two-dimensional scan cannot assess bone structural adaptations to exercise that influence bone strength. Three-dimensional imaging tools such as high-resolution peripheral quantitative computed tomography (HR-pQCT) have evolved to demonstrate compartment- (compact and trabecular bone) and strength-specific adaptations of growing bone. To our knowledge, this instrument has never been used to assess bone’s response to exercise in children with JIA.

Our study in children with JIA had three objectives: 1. to evaluate safety and feasibility of a targeted 6-month home-based exercise program, 2. to estimate effect of the exercise program on bone mass (by DXA), structure and strength (by HR-pQCT), muscle function and clinical outcomes, 3. to assess stability of bone and muscle outcomes 6 months after the intervention. Results will be used to inform design and implementation of larger randomized controlled trials.

## Methods

We conducted a pre-post exercise intervention trial with children and youth with JIA in Vancouver, Canada. This sub-study was part of a large multisite, longitudinal, observational cohort study of children with JIA (LEAP; Linking Exercise, Physical Activity and Pathophysiology in Childhood Arthritis). LEAP aims to investigate the relationships between JIA, PA and bone and muscle development (http://www.leapjia.com/).

### Recruitment

Between September 2014 and February 2015, we recruited children aged 8 to 16 years with a diagnosis of JIA [21] who were willing and able to participate, irrespective of their subtype of JIA or disease activity status, and who lived in the Metro Vancouver area and could attend monthly group exercise sessions. Exclusion criteria were: 1. receiving bisphosphonate treatment (past or planned), 2. participation in high performance sports, training or competition > 3 h/week, 3. participation > 1 resistance training session per week for the past 4 months, and 4. pregnant or planning pregnancy. The Clinical Research Ethics Boards at the University of British Columbia approved all procedures (certificate H14–01572). Parents or guardians provided written informed consent and children provided assent.

### Measurement

Following recruitment, participants attended baseline measurement at the Centre for Hip Health and Mobility in Vancouver. To account for rolling recruitment the intervention was conducted in three waves (February, March and April, 2015). Participants had follow-up assessments 3, 6 and 12 months after their baseline visit. At each assessment participants completed questionnaires, muscle testing and clinical assessment by a pediatric rheumatologist. Bone outcomes were assessed at baseline, 6- and 12-months.

Clinical assessment included count of joint and enthesitis sites with active inflammation by a pediatric rheumatologist, standing and sitting height using stretch stature (Seca Model 242, Hanover, MD) to the nearest 0.1 cm, body weight calibrated electronic scale (Seca Model 840, Hanover, MD) to the nearest 0.1 kg and arm (ulna) and lower leg (tibia) length (cm) to the nearest millimeter using a standard steel anthropometric tape. All measures were performed in duplicate by trained research assistants.

We estimated age at peak height velocity (APHV) as an indicator of somatic maturation using the Moore equation [22], and subsequently calculated maturity offset as a continuous measure of years from APHV. Self-report questionnaires included pain intensity in last week (10 mm visual analog scale where higher scores indicate more pain), function (Child Health Activity Questionnaire (CHAQ), 0–3 where higher scores indicate greater functional disability) [23], physical activity questionnaire for children (PAQ-C) and adolescents (PAQ-A) (score 1–5 with higher scores indicating higher PA) [24, 25], fatigue (PedsQL Multidimensional Fatigue Scale, 0–100 where higher scores indicate less fatigue) [26], Juvenile Arthritis Quality of Life Questionnaire (JAQQ, scores 1–7 with lower scores indicated better quality of life) [27], Child and Youth Physical Self-perception Profile (CYPSP, 4 sub-domains of sport competence, body attractiveness, physical condition, and physical strength as well as general physical self-worth; score 0–4 with 2.5 midpoint and higher scores indicating higher self-perception) [28], current medications, fracture history, dietary calcium intake [29] and self-assessed stage of sexual maturation as per the method of Tanner [30].

We used both DXA and HR-pQCT to assess bone outcomes at baseline, 6- and 12-months. Image acquisition and analysis protocols are described in detail elsewhere [31]. Lumbar spine bone mineral content (BMC, g) and areal bone mineral density (aBMD, g/cm^2^) were assessed using DXA (Hologic QDR 4500 W) with standard positioning. A trained technician acquired and analyzed all scans and performed daily quality control procedures. We calculated lumbar spine BMC z-scores using published reference data [32]. Second, we used HR-pQCT (XtremeCT; Scanco Medical AG, Brüttisellen, Switzerland) to assess BMD, bone microarchitecture and bone strength at the non-dominant distal radius (7% site) and distal tibia (8% site). Outcomes of interest included total BMD (Tt. BMD, mg HA/cm^3^), trabecular bone volume ratio (BV/TV), cortical thickness (Ct.Th, mm; mean cortical volume divided by the outer bone surface) and failure load (F.Load, N). We calculated age-, sex- and ethnicity-specific z-scores for each HR-pQCT outcome using published centile values [31].

Muscle testing included grip strength, peak power (single two-legged countermovement vertical jump) and force (multiple one-legged hopping) on Leonardo Mechanograph® Ground Reaction Force Plate, (GRFP; Novotec Medical GmbH, Germany), and isokinetic strength of non-dominant elbow flexors / extensors, knee flexors / extensors, hip abductors/ adductors using a Biodex dynamometer (System 4). We highlight grip strength, peak power, knee extensor peak torque, average power and total joint work per participant as clinically significant muscle measures.

### Exercise intervention

The home-based exercise program included: jumping exercises (3 sessions/week, jumps associated with ground reaction forces of between 3 and 5 times body weight [33, 34]); handgrip exercises (3 sessions/week completed with hand grippers) and resistance training (2 sessions/week completed with Therabands® without handles). Before starting the program, our kinesiologist (KD) conducted a home visit to individually tailor the program to each participant’s abilities. The kinesiologist provided each participant with pictures of their exercises, a schedule for exercise progression, Therabands® (color coded for resistance) and hand grippers. She also demonstrated modified exercises should specific exercises cause joint pain and recommended one rest day between resistance and jumping exercises.

The progressive exercise program was implemented over 26 weeks. Participants completed six 4-week blocks of jumping exercises (exercise load increased from 1 set of 5 jumps to 3 sets of 10 jumps; jumping difficulty progressed from simple jumping movements (e.g., tuck jumps/‘motorcycle jumps’) to hopping, skipping and multidirectional plyometric movements (e.g. jumping lunge/‘leaping lizards’) Additional file 1: Figure S1). We estimated ground reaction forces associated with these exercises were approximately 3 to 5 times body weight [35]. For resistance exercises the load increased from 1 set of 8 repetitions for 4 exercises to 3 sets of 15 repetitions for 7 exercises. Difficulty increased from simple single-joint exercise (e.g. biceps curl) to complex multiple- exercises (e.g. lunges). Participants were instructed to take a 20-s rest break between sets and exercises. Resistance exercises included upper extremity, core and lower extremity muscle groups. Handgrip exercises progressed from 2 sets of 5 repetitions to 3 sets of 12 repetitions. Children were instructed to squeeze a rubber cylinder with either 5 short, hard squeezes or hold a moderate to maximal squeeze for 5 s. We implemented a 2-week program of exercise maintenance at the end of the six 4-week blocks. The resistance training exercises required 10–30 min per session; jumping and handgrip exercises required 2–10 min per session.

Participants also attended one group-based exercise session each month. These kinesiologist-led sessions were held at three locations in Metro Vancouver as per the geographic distribution of participants. At each session, the kinesiologist reviewed participants’ progress, introduced new exercises, conducted interactive strength and agility activities and provided children with the opportunity to meet fellow participants. The kinesiologist modified the exercise program for participants who reported pain during or after completion of prescribed exercises.

Participants received usual medical care throughout the study and had regular rheumatology clinic visits every three to 4 months. If clinically indicated, clinic visits included assessment by a physiotherapist who could prescribe additional exercises to target specific problem areas.

### Monitoring / adherence

Participants completed a weekly exercise log, in print or online. (Additional file 1: Figure S2). Parents/guardians were asked to assist with scheduling and monitoring home exercise sessions, as needed. The kinesiologist contacted the family every 2 weeks to address questions about exercises and to monitor compliance. We recorded the proportion of prescribed exercise completed (total repetitions reported / total repetitions prescribed) and the proportion of group exercise sessions attended (sessions attended/total sessions). We also asked participants to use their log book to record completion of any additional exercises.

### Safety and feasibility

Adverse events included JIA flare (defined as any increase in active joint count or Physician Global Assessment of disease activity that resulted in a change in treatment), post-exercise pain lasting > 24 h, and falls or injuries that occurred during exercise. To determine feasibility, we recorded participant recruitment, adherence to exercises, attendance at group exercise sessions, completion of questionnaires and attendance at measurement session. As a companion study, we conducted one-on-one interviews with child-caregiver dyads to identify facilitator and barriers to adherence [36].

### Sample size

We recruited a convenience sample of 24 participants as this allowed 8 subjects per group for each recruitment wave.

### Statistical analysis

We performed all analyses using Stata, version 10.1 (StataCorp, College Station, TX). We report means and standard deviations for anthropometry, clinical, bone and muscle outcomes. We report median and interquartile range (IQR) for categorical outcomes, including active joint count. We calculated the average (and range of) adherence for each 4-week exercise block and the proportion of participants attaining at least 80% adherence for each 4-week block. We used repeated-measures analysis of variance to compare clinical outcomes over time. To examine change in bone and muscle outcomes while accounting for the time-varying covariate change in height, we fit mixed effects models (*xtmixed* in Stata) with time as a fixed effect and maturity offset at baseline as an additional covariate. We used the *margins* command in Stata for pairwise comparisons between time points. We considered *p* values < 0.05 as statistically significant.

## Results

**Fig. 1:**
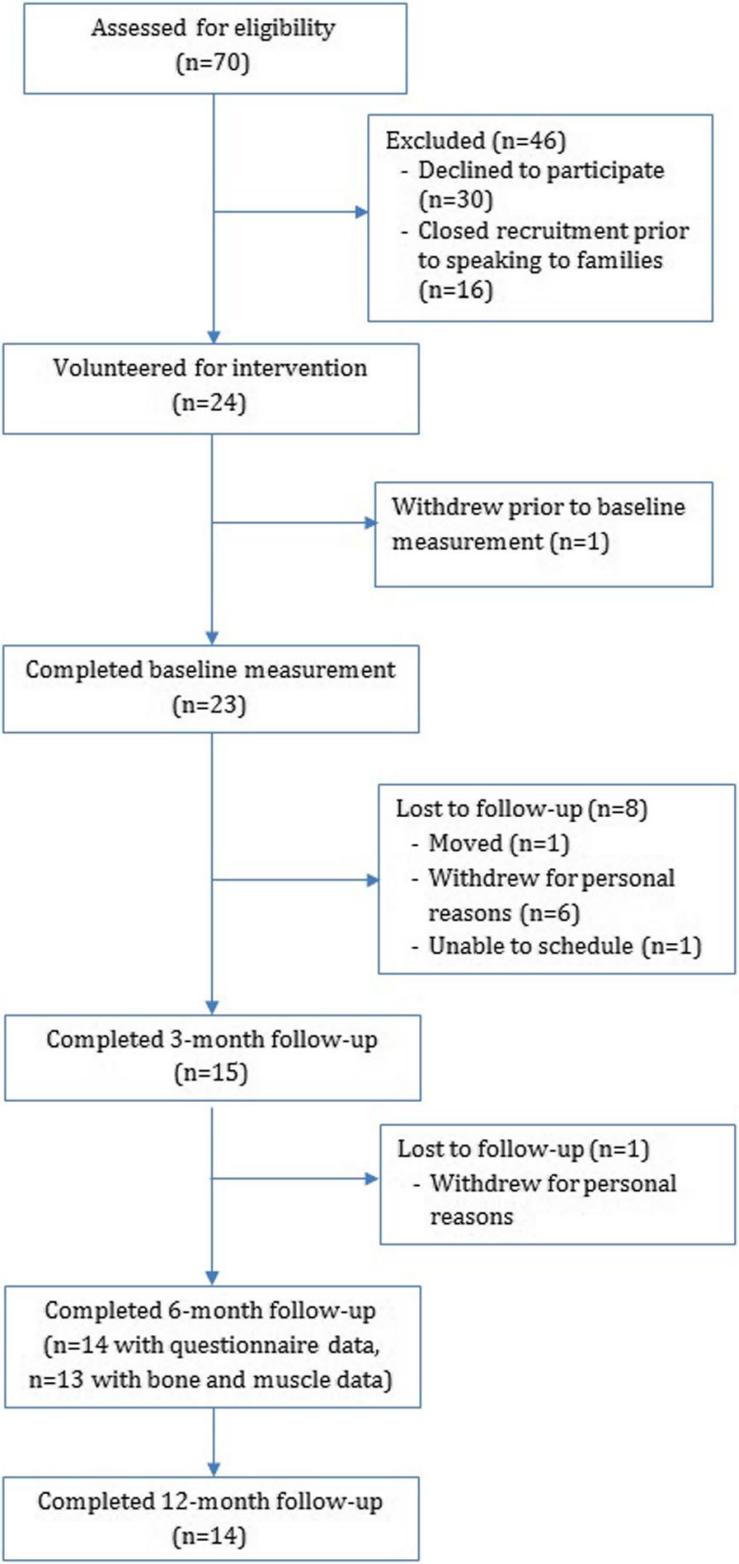
Flow of participants through the trial

We summarize flow of participants through the trial in Fig. 1. Twenty-four participants volunteered to participate out of 54 approached (44%). Those declining participation expressed no interest or lack of time. Twenty-three participants completed baseline assessment. Thirteen participants (56%) completed baseline, 6- and 12-month measures. Participants who withdrew were similar in age, height, weight and clinical characteristics to participants who completed the study (Additional file 2: Table S1).

In Table 1, we summarize descriptive characteristics at baseline for those who completed the study. Most children (62%) were Caucasian and pre- or early pubertal. Most participants had a healthy BMI (*n* = 9, 6 girls), 1 boy was underweight, 1 girl and1 boy were overweight and 1 boy was obese, as per WHO criteria [37]. Most participants (77%) had oligoarticular disease; enthesitis-related arthritis was the most common JIA subtype in boys (*n* = 3, 50%). At baseline, 7 children were on non-steroidal anti-inflammatory drugs, 9 were on disease modifying therapy and 4 were on biologic therapy. Medication details for each participant are provided in Additional file 2: Table S2. Most participants had inactive disease at baseline; however, function, as measured by the CHAQ, was impaired in 6 participants (46%) (Table 2). Average PA score was low to average in girls and boys; general physical self-worth was higher than the midpoint score of 2.5 in both sexes.

**Table 1:** Descriptive characteristics at baseline of participants who completed the study (*n* =!3). Values are mean (standard deviation) unless otherwise indicated

Variable	All participants (*n* = 13)	Girls (*n* = 7)	Boys (11 = 6)
Age (years)	13.0(2.0)	13.5 (2.3)	12.4(1.6)
Ethnicity (tt Asian/Caucasian/ Other)	3/8/2	1/5/1	2/3/ 1
Tanner stage (# 1/2/3/4/5)	3/3/4/2/1	1 /2/3/1/0	2/ 1/1/ 1/1
Maturity offset (years)	0.07(1.8)	1.0 (1.9)	−1.0 (1.0)
Height (cm)	149.7(8.3)	150.0(9.6)	149.4 (7.4)
Height z-score	−0.61 (0.83)	−0.80(0.%)	−0.39 (0.66)
Weight (kg)	42.6(7.0)	41.1 (43.9)	41.1 (5.0)
Weight z-score	−0.39 (1.26)	−0.48(1.21)	− 0.28 (1.42)
BMI (kg/m^2^)	18.8(2.6)	19.4 (2.2)	18.5(3.1)
BMI z-score	−0.07 (1.26)	0.02(0.87)	−0.19 (1.69)
Clinical outcomes			
Time since diagnosis (months)	57 (47)	78 (23)	34 (49)
JIA subtvpes - polyarticular course	4 (2 poly RF negative, 2 oligo extended)	3 (2 poly RF negative, 1 oligo extended)	1 (oligo extended)
JIA subtypes - oligoarticular course	9 (2 oligo persistent. 3 ERA. 2 psoriatic, 2 undi fterentiated)	4 (2 psoriatic, 1 oligo persistent, 1 undifferentiated)	5 (3 ERA, 1 oligo persistent, 1 undifferentiated)
Medications (n)	7 NSAIDS,9 DMARDs, 4 Biologies	5 NSAIDS, 6 DMARDs, 3 biologies	2 NSAIDS, 3 DMARDs, 1 biologic
Fracture history	4	1 distal radius, 1 nasal	1 distal radius, 1 distal tibia
Dietary calcium intake (mg)	740 (410)	468 (196)	1058 (364)

NSAIDs non-steroidal anti-inflammatory medications including Naproxen. Ibuprofen. DMARDs, Disease modifying anti-rheumatic drugs including Methotrexate, Leflunomide, Sulfasalazine. Biologies - including Etanercept. Infliximab, Humira

**Table 2:** Clinical measures for group at baseline, 3- and 6- and 12-months. Values are mean (standard deviation) unless otherwise indicated. *P*-value indicates the main effect of time from the repeated measures analysis of variance

Clinical measure	Baseline	3-months	6-months	12-months	p-value
Median active joint count^a^	0 (0, 0) range 0–1	0 (0,0.5) range 0–7	0 (0, 0) range 0–1	0 (0,0) range 0–2	
Median restricted joint count	0 (0, 0) range 0–3	0 (0,0) range 0–13	0 (0, 0) range 0–3	0 (0,0) range 0–1	
Median active enlhesitis count	1.0 (1.5)	0 (0.0) range 0–3	0 (0.0) range 0–0	0 (0.0.5) range 0–4	
Functional ability (CHAQ, range 0–3)	0.20 (0.42)	0.38 (0.54)	0.46 (0.53)	0.33 (0.48)	0.216
Pain (VAS, range 0–100)	26.0 (26.6)	15.5 (20.8)	25.4 (20.4)	18.0(22.5)	0.334
JAQQ score (range 1–7)	2.6 (1.0)	2.3 (1.2)	2.3 (1.0)	2.4 (1.2)	0.384
PA Score (range 1–5)	2.3 (0.6)	2.6 (0.72)	2.2 (0.5)	2.3 (0.9)	0.469
Fatigue (PedsQL. range 0–100)	57.0 (18.2)	66.0 (18.0)	69.9 (18.9)	67.3 (17.2)	0.034b
Physical self-worth (CYPSPP. range 0–4)	3.3 (0.6)	3.2 (.6)	3.3 (0.7)	3.4 (0.6)	0.966

*CHAQ* Child Health Assessment Questionnaire, *VAS* visual analog scale, *JAQQ* Juvenile Arthritis Quality of Life Questionnaire, *PA* physical activity score from the Physical Activity Questionnaire for Children (8–14 y) and Adolescents (14–18 y), *PedsQL* Pediatric Quality of Life Inventory, *CYPSPP* Child and Youth Physical Self-perception Profile

^a^Most participants had 0 active joints. At baseline 1 participant had 1 active joint. At 3 months, 1 patient had 7 active joints. At 6 months, one participant had 2 active joints and 1 had 3 active joints. At 12 months, 1 participant had 1 active joint and 1 had 2 active joints. b *p* < 0.05

### Adherence

Participants completed a median of 46.9% (5.4, 66.7 IQR) of prescribed exercises. Adherence was highest during the first 4-week block of exercises (median 72.3%). It decreased but remained stable over the next 16 weeks (approximately 50%) and decreased during the final 4 weeks (median 38%). Attendance at monthly group sessions was variable across the intervention period, with a median of 66.7% (16.7, 100 IQR) sessions attended. Only 2 participants (15.4%) achieved the goal of 80% adherence, 3 (23.1%) achieved 60% adherence and 5 (38.5%) achieved 50% adherence. Completion of logbooks (paper or online) was 53.8% (19.2, 91.7 IQR) and declined across 24-weeks.

### Pain, injury and flare

Pain scores were variable and generally low (27 ± 26 points) on 100-point scale (Table 2, Additional file 2: Table S2). Ten incidents of pain lasting > 24 h were reported by 8 participants. The incremental change in pain from pre- to post-exercise sessions was small (5.0, 0–20 Median, IQR on 100-point scale). Two participants reported falls or injuries; one participant ‘rolled their ankle’ during the jumping exercises and one participant reported a knee injury in two consecutive jumping exercises sessions. Seven participants had their exercise programs modified for pain, injury or flare, and 3 withdrew because of pain. There were 4 JIA flares during the intervention (3 mild, 1 moderate). Of those participants with mild flares, one participant (#16) was previously in clinical remission and started NSAID therapy and two participants (#9, #19) were previously in clinical remission on stable DMARD therapy but started NSAID therapy during the intervention. One participant (#8) had a moderate flare that required addition of prednisone and biologic therapy to stable DMARD therapy.

### Clinical outcomes

**Fig. 2:**
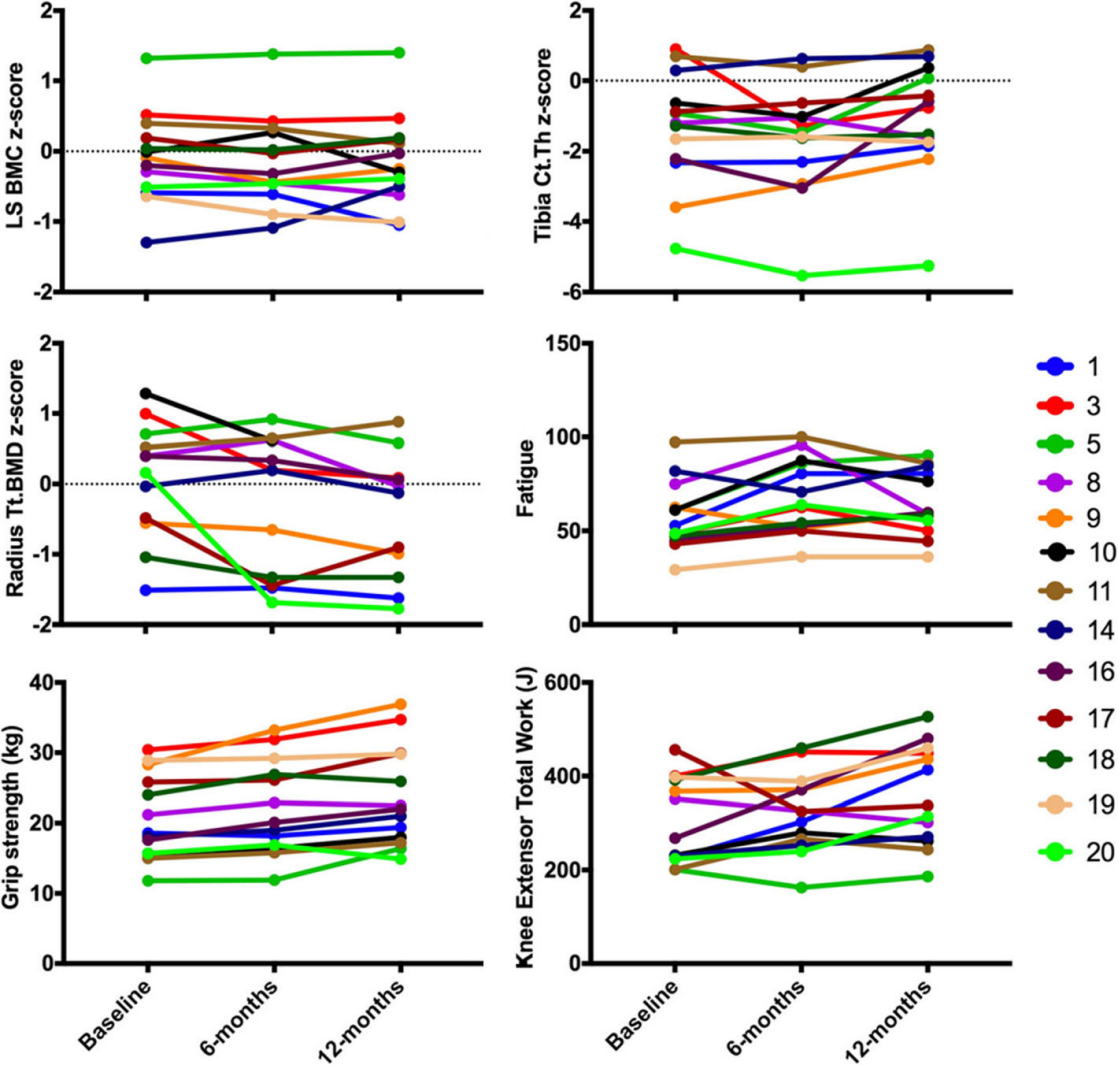
Individual time series plots for the 13 participants that completed the 6-month intervention and 6-month follow-up. The plot for total bone mineral density (Tt.BMD) z-score at the distal radius does not include data for participant 19 due to significant motion artifact present in the baseline scan

Total fatigue scores were low at baseline (mean 57.9, SD 18.2) and improved by 8.1 points and 12.0 points, on average, at 3 and 6 months, respectively, with some improvement maintained at 12 months (9.4 points) (Table 2, Fig. 2). Total fatigue scores were lower than previously reported scores of a general pediatric rheumatology population aged 2 to 18 years (mean 73.8, SD 21.9) [26]. We did not observe improvements in any other clinical outcomes.

### Bone outcomes

We provide bone outcomes at baseline, 6- and 12-months in Table 3. We excluded one participant’s baseline radius HR-QCT scan because of significant motion artifact (motion grade of 5 on a 5-point scale). We identified one potential outlier for HR-pQCT outcomes at 12-months. Distal radius Z-scores for this participant at 12-months were more than 2 standard deviations higher than their z-scores at baseline or 6-months and review of the participant’s scan revealed callus formation of unknown origin (no history of fracture). We removed the 12-month HR-pQCT scan for this participant from our analysis.

**Table 3:** Bone outcomes at baseline, 6- and 12-months of those participants who completed the study (*n* = 13). Values are mean (standard deviation) at baseline and 6- and 12-months and adjusted mean change (95% confidence interval) from baseline to 6-months and 12-months

Variable	Baseline	6-months	12-months	Baseline – 6-months change^d^	Baseline – 12-months change^d^
LS BMC (g)	32.6 (8.4)	35.0 (8.3)	37.0 (9.1)	0.3 (− 0.9, 1.6)	1.2 (− 0.5, 2.8)
LS BMC z-score	−0.09 (0.64)	− 0.14 (0.65)	− 0.14 (0.65)	− 0.04 (− 0.24, 0.16)	−0.02 (− 0.28, 0.23)
Distal tibia Tt.BMD (mg/cm^3^)	223.1 (34.1)	223.8 (28.7)	234.9 (29.5)	2.7 (−5.0, 10.5)	15.0 (5.0, 25.0)^a^
z-score	− 0.98 (1.73)	− 1.10 (1.47)	− 0.86 (1.60)	− 0.15 (− 0.44, 0.13)	0.06 (− 0.30, 0.43)
# with z-score < − 2	3	2	3		
Distal tibia BV/TV	0.141 (0.026)	0.141 (0.022)	0.144 (0.026)	−0.001 (− 0.006, 0.004)	0.001 (− 0.005, 0.007)
z-score	− 0.74 (1.48)	− 0.79 (1.30)	− 0.69 (1.46)	− 0.08 (− 0.31, 0.15)	0.01 (− 0.29, 0.30)
# with z-score < − 2	4	3	3		
Distal tibia Ct.Th (mm)	0.69 (0.15)	0.69 (0.17)	0.77 (0.14)	−0.002 (− 0.054, 0.050)	0.075 (0.009, 0.142)^b^
z-score	−1.35 (1.62)	− 1.65 (1.59)	− 1.08 (1.62)	− 0.62 (− 1.14, − 0.10)^d^	−0.23 (− 0.91, 0.44)
# with z-score < − 2	4	4	2		
Distal tibia F.Load (N)	3628 (845)	3825 (771)	4121 (878)	4.8 (− 204.9, 214.6)	190.2 (−81.1, 461.5)
z-score	−1.57 (1.58)	− 1.58 (1.14)	− 1.33 (1.39)	−0.08 (− 0.40, 0.25)	0.13 (− 0.29, 0.55)
# with z-score < − 2	4	4	4		
Distal radius Tt.BMD (mg/cm^3^)^c^	251.3 (37.7)	245.5 (29.4)	242.3 (33.8)	−0.5 (− 14.2, 13.1)	1.5 (− 16.6, 19.6)
z-score	0.07 (0.83)	−0.26 (0.99)	− 0.47 (0.90)	− 0.42 (− 0.80, − 0.03)^d^	−0.60 (− 1.11, − 0.09)^b^
# with z-score < − 2	0	0	0		
Distal radius BV/TV^c^	0.136 (0.022)	0.139 (0.023)	0.135 (0.027)	0.002 (−0.004, 0.007)	− 0.004 (− 0.011, 0.004)
z-score	−0.50 (0.97)	− 0.42 (1.01)	−0.67 (1.32)	0.01 (− 0.27, 0.30)	−0.27 (− 0.64, 0.10)
# with z-score < − 2	1	1	1		
Distal radius Ct.Th (mm)^c^	0.62 (0.15)	0.60 (0.13)	0.60 (0.16)	0.03 (−0.03, 0.10)	0.08 (− 0.01, 0.16)
z-score	−0.21 (1.01)	− 0.61 (0.98)	−0.77 (0.83)	− 0.36 (− 0.97, 0.24)	−0.39 (− 1.18, 0.40)
# with z-score < − 2	0	1	0		
Distal radius F.Load (N)^c^	1217 (392)	1297 (393)	1301 (375)	27.6 (−43.7, 99.0)	− 10.2 (− 105.3, 84.8)
z-score	−0.93 (1.47)	− 1.02 (1.32)	−1.25 (1.37)	− 0.22 (− 0.55, 0.12)	−0.38 (− 0.83, 0.06)
# with z-score < − 2	3	2	3		

*BMC* bone mineral content, *Tt.BMD* total bone mineral density, *BV/TV* trabecular bone volume ratio, *Ct.Th* cortical thickness, *F.Load* failure load

^a^*p* < 0.01; ^b^*p* < 0.05

^c^*n* = 12 at baseline and 6-months and *n* = 11 at 12-months

^d^ Adjusted for change in height and maturity offset at baseline

At baseline, all participants had a LS BMC z-score within the normal range as compared with international reference data [32]. In contrast, 4 participants had z-scores < − 2 for HR-pQCT outcomes at the distal tibia (Tt.BMD, BV/TV, Ct.Th and F.Load). Three of these participants also had z-scores < − 2 for F.Load and BV/TV at the distal radius.

LS BMC z-score did not change over the course of the intervention or follow-up period (Table 3, Fig. 2). At the distal tibia, Ct.Th z-score appeared to decrease during the intervention; however, this trend was not maintained after 12-months. Total BMD at the distal tibia increased by 5% after 12 months, but Tt.BMD z-score did not change significantly. Z-scores for distal tibia BV/TV and F.Load did not change significantly. At the distal radius, Tt.BMD z-score decreased during follow-up after adjusting for change in height and maturity. Z-scores for other bone outcomes at the distal radius did not change significantly over time.

### Muscle outcomes

We provide key muscle outcomes at baseline, 3-, 6- and 12-months in Table 4. Briefly, after adjusting for height and maturity offset, grip strength and knee extensor peak torque, average power and total joint work did not improve over the course of the intervention (Table 4, Fig. 2). Absolute values for peak force of the right and left legs increased between baseline and 12-months, but z-scores for mechanography outcomes did not change significantly during the intervention.

**Table 4:** Muscle outcomes assessed by dynamometry and mechanography in those participants that completed the study (*n* = 13). Values are mean (standard deviation) at baseline, 3-, 6- and 12-months and adjusted mean change (95% confidence interval) for change from baseline to 3-, 6- and 12-months

Variable	Baseline	3-months	6-months	12-months	Baseline-3 months^c^	Baseline-6 months^c^	Baseline-12 months^c^
Grip strength, left (kg)	19.5 (6.3)	19.6 (6.1)	19.8 (6.8)	21.6 (6.1)	−0.8 (−2.2, 0.6)	−1.5 (−3.2, 0.2)	−0.7 (2.9, 1.5)
Grip strength, right (kg)	20.9 (6.0)	22.3 (7.5)	22.0 (6.6)	23.7 (.1)	0.6 (−0.6, 1.9)	−0.4 (−1.9, 1.0)	0.5 (−1.4, 2.4)
Peak power (W/kg)	37.8 (5.5)	39.3 (7.3)	38.2 (5.5)	39.3 (5.9)	0.8 (−1.1, 2.7)	−0.9 (−3.3, 1.4)	− 0.7 (− 3.6, 2.3)
Peak power z-score	− 0.79 (0.95)	−0.58 (0.98)	− 0.84 (0.94)	−0.70 (0.91)	0.13 (− 0.21, 0.47)	−0.20 (− 0.61, 0.21)	−0.15 (− 0.67, 0.37)
Peak force, right leg (F_max_/BW)	2.84 (0.40)	2.82 (0.39)	2.80 (0.33)	2.90 (0.33)	−0.00 (− 0.01, 0.01)	−0.01 (− 0.02, 0.00)	0.21 (0.19, 0.22)^a^
Peak force, right leg z-score	−0.82 (1.18)	− 0.52 (1.30)	−0.95 (1.16)	− 0.63 (1.07)	0.31 (− 0.14, 0.75)	−0.12 (− 0.66, 0.41)	0.21 (− 0.48, 0.89)
Peak force, left leg	2.75 (0.42)	2.94 (0.41)	2.74 (0.31)	2.80 (0.36)	−0.01 (− 0.02, 0.00)	−0.02 (− 0.03, − 0.003)^b^	0.19 (0.17, 0.21)^a^
Peak force, left leg z-score	− 1.15 (1.36)	−0.88 (1.25)	−1.16 (1.06)	− 1.02 (1.28)	0.15 (− 0.29, 0.59)	−0.23 (− 0.78, 0.30)	−0.23 (− 0.91, 0.45)
KE Peak torque (N-m)							
60 °·s 1	182.0 (29.4)	192.5 (38.9)	203.4 (53.1)	219.7 (65.5)	−3.4 (−21.9, 15.0)	−8.6 (− 31.3, 14.0)	− 5.8 (− 33.9, 22.3)
180 °·s 1	145.1 (27.3)	160.9 (29.5)	167.7 (34.7)	181.3 (49.5)	7.3 (− 4.5, 19.0)	4.5 (−9.9, 19.0)	9.6 (−8.4, 27.6)
KE Average power (W)							
60 °·s 1	44.1 (11.2)	50.3 (15.2)	53.1 (17.4)	56.3 (22.7)	0.1 (−6.5, 6.7)	−3.5 (−11.6, 4.6)	− 6.7 (− 16.8, 3.3)
180 °·s 1	91.5 (24.6)	105.6 (25.7)	107.4 (27.1)	112.0 (31.6)	7.0 (−2.4, 16.4)	2.1 (−9.4, 13.6)	−1.4 (− 15.7, 12.9)
KE Total joint work (J)							
60 °·s 1	303.5 (91.8)	318.0 (76.6)	322.5 (89.1)	360.7 (107.3)	−4.8 (−38.6, 29.0)	− 18.8 (− 60.3, 22.6)	−2.9 (− 54.3, 48.4)
180 °·s 1	571.5 (162.1)	635.8 (154.1)	639.9 (156.7)	667.8 (170.2)	31.0 (−26.4, 88.5)	4.2 (−66.2, 74.7)	−7.3 (− 94.8, 80.1)

*KE* knee extensor; ^a^*p* < 0.001; ^b^ p < 0.05; ^c^ adjusted for change in height and maturity offset at baseline

## Discussion

We address an important knowledge gap in the area of exercise therapy for children with JIA and extend the current literature in five ways. First, results of our intervention trial suggest it is safe to engage children with JIA in a home-based exercise program, designed to enhance muscle and bone strength. Second, participation and adherence were low, suggesting the exercise program was not feasible to extend to the larger JIA population. Third, fatigue improved among those participants who completed the study, and was sustained 6 months after cessation of the intervention. Fourth, we used standard clinical DXA to assess change in bone mass and an advanced imaging tool, HR-pQCT to assess change in bone microstructure, BMD and estimated bone strength. Fifth, and finally, we used several techniques to assess muscle function that were feasible for children with JIA to perform.

### Safety and feasibility

Our exercise program is the first to include impact exercise and resistance training in both home- and group-based sessions. As noted by others [6], a ‘one size fits all’ approach to exercise prescription is likely not appropriate for children with JIA. Thus, our kinesiologist tailored the program to each participant’s abilities and modified the program when necessary. Modifications were made for pain, injury or flare in seven participants. Adverse events and post-exercise pain were rare, which suggests the exercise program is safe for children with JIA.

The six-month intervention was necessarily longer (to promote osteogenesis) than previous studies that employed resistance training and weight-bearing exercise to promote bone strength [19, 38]. The longer duration may have contributed to high participant attrition and relatively low adherence. Some participants expressed lack of motivation, which also emerged as a recurring theme in our companion study where we used qualitative methods to better understand barriers to participating in exercise among children with JIA [36]. Previous studies also reported low adherence to exercise programs and physical therapy among children with JIA [39–41]. However, even partial adherence to treatment plans may significantly decrease pain and improve function and quality of life [39].

Our study demonstrates a clear need to better understand barriers and facilitators to participation in and adherence to exercise programs in children with JIA. In future, the exercise intervention could be adapted to include more group- or in person-contact with the exercise instructor, incentives for participation, or incorporate interactive technologies (e.g. Fitbits), social media (e.g. Strava) or internet-based interventions to monitor and encourage adherence, particularly when participants are not in close geographic proximity [42–44]. Armbrust and colleagues recently reported promising results for their internet-based PA intervention, “Rheumates @ Work” (R@W) [45]. In contrast to the present study, R@W did not include a prescriptive exercise component. Instead, each week of the interactive, cognitive-behavioral program targeted a different theme such as health education and barriers and benefits of being physically active through online films, animations, puzzles and/or assignments. Acceptance and satisfaction with R@W were very high (93.8% and 85%, respectively) among children aged 8 to 13 years [44] and this translated into improvements in children’s PA and exercise capacity [45]. Further, low program costs suggest this might be a feasible program for others to implement, perhaps in combination with recommended activities to promote muscle and bone strength, rather than a prescriptive exercise component.

### Clinical outcomes

Participants had low disease activity for the duration of the study, but almost half reported some functional difficulties (CHAQ above 0). PA levels were low to average and did not change during the study. Fatigue was common but diminished after 6 months of exercise; this decrease was sustained at 12 months. This is promising as fatigue may be a barrier to participation in PA and organized sport among children with JIA [46]. We did not observe any other improvements in clinical outcomes, which may in part relate to low adherence.

### Bone and muscle outcomes

Ours is the first study to utilize HR-pQCT to examine bone structure and strength in children with JIA. Of note, whereas DXA-derived lumbar spine BMC z-scores were within the normal range for all participants, several participants had z-scores for bone strength, structure and density lower than two standard deviations at both the distal tibia and radius. Similar to previous studies that employed pQCT imaging [15–17], this finding suggests that bone strength and structure may be compromised in children with JIA. We aim to investigate this further in the larger LEAP study.

The intervention did not positively influence children’s bone mass, structure or strength z-scores. Although short bouts of jumping activity are known to benefit bone structure at the weight-bearing tibia in school-aged children without JIA [34, 47], low adherence and attrition (implementation factors) likely contributed to our inability to adequately assess effect of the intervention itself (design factors) on bone mass, structure and strength in children with JIA. Further, 6 months may have been insufficient to accurately assess exercise-related adaptations in these bone outcomes. The decline in Tt.BMD z-score at the distal radius may also suggest that modifications to the exercise program are required to provide a more osteogenic stimulus (e.g., dynamic loads of greater magnitude) to the upper limbs.

We extend previous studies of children with JIA that measured strength for single muscle groups, by incorporating jumping mechanography to assess peak force and peak power [48]. Muscle function measured by mechanography is not well described in children with arthritis. However, these measures may be more relevant to children’s daily activities than traditional single muscle or muscle group strength testing. Children with JIA performed jumping mechanography without difficulty and demonstrated modest improvements in muscle function over the course of the study. However, changes in z-scores for mechanography outcomes as well as grip strength and knee extensor torque, power and total work were likely a function of normal growth and development as the increases over 3-, 6- and 12-months were no longer significant after we accounted for height and maturity.

We acknowledge several limitations of our study. Intervention efficacy was hampered by relatively high attrition and low adherence. It will be important in future to identify factors that negatively impact implementation and overcome them, where possible. Also, we did not have a control group with which to compare change in outcomes and accurately tease out the distinct influences of growth and maturation separate from the intervention on bone and muscle outcomes. Finally, we did not assess serum levels of vitamin D nor did we provide participants with calcium (or vitamin D) supplements despite the fact that most participants did not meet the dietary reference intake for calcium of 1300 mg/day [49]. Supplementation may be warranted in future exercise trials. Further, the influence of dietary calcium intake on bone outcomes in children with JIA will be explored in the larger LEAP study.

## Conclusions

Children with JIA safely participated in a 6-month targeted home- and group-exercise program that incorporated short bouts of high-impact exercise and resistance training to increase bone and muscle strength. We highlight key challenges of exercise interventions in this population including high attrition and low adherence. There is a sensitive balance between designing an effective exercise program to promote children’s muscle and bone health and implementing it effectively in children with JIA. Future studies should devise strategies that mitigate pain and barriers to implementation, including novel approaches to enhance compliance and minimize attrition. Examples may include innovative program delivery via web-based online platforms and more effective participation incentives using customized apps and wearable technology. Promotion of physical literacy and age appropriate physical activity, including recommendations of activities that strengthen muscle and bone at least 3 days per week, may be more effective than precise exercise prescription. Continued efforts are needed to encourage a physically active lifestyle as a means to promote overall health and well-being in children with JIA.

## Additional file

Additional file 1: **Figure S1.** Exercise Log. (JPG 189 kb)

Additional file 2: **Table S1.** Comparison of descriptive and clinical outcomes, lumbar spine bone mineral content (BMC) z-score and total bone mineral density (Tt.BMD) at the distal tibia between those participants that withdrew and those that completed all assessments. Values are mean (standard deviation) unless otherwise indicated. **Table S2.** Clinical outcomes at baseline, 3-, 6- and 12-months for each participant who completed the study (n=13). (DOCX 34 kb)

## Abbreviations

APHV: Age at peak height velocity
BMC: Bone Mineral Content
BMD: Bone Mineral Density
BV/TV: Trabecular bone volume ratio
CHAQ: Child Health Activity Questionnaire
Ct.Th: Cortical thickness
CYPSP: Child and Youth Physical Self-perception Profile
DXA: Dual energy X-ray absorptiometry
F.Load: Failure load
HR-pQCT: High-resolution peripheral quantitative computed tomography
JAQQ: Juvenile Arthritis Quality of Life Questionnaire
JIA: Juvenile idiopathic arthritis
LEAP: Linking Exercise, Physical Activity and Pathophysiology in Childhood Arthritis
MVPA: Moderate to vigorous physical activity
PA: Physical activity
PAQ-A: Physical activity questionnaire - adolescents
PAQ-C: Physical activity questionnaire – child

## References

[1] Bos GJFJ, Lelieveld OTHM, Armbrust W, Sauer PJJ, Geertzen JHB, Dijkstra PU (2016). Physical activity in children with juvenile idiopathic arthritis compared to controls. Pediatr Rheumatol Online J.

[2] Lelieveld OTHM, Armbrust W, van Leeuwen MA, Duppen N, Geertzen JHB, Sauer PJJ (2008). Physical activity in adolescents with juvenile idiopathic arthritis. Arthritis Rheum.

[3] van Brussel M, Lelieveld OTHM, van der Net J, Engelbert RHH, Helders PJM, Takken T (2007). Aerobic and anaerobic exercise capacity in children with juvenile idiopathic arthritis. Arthritis Rheum.

[4] Cavallo S, April KT, Grandpierre V, Majnemer A, Feldman DE (2014). Leisure in children and adolescents with juvenile idiopathic arthritis: a systematic review. PLoS One.

[5] Cavallo S, Majnemer A, Duffy CM, Feldman DE (2015). Participation in leisure activities by children and adolescents with juvenile idiopathic arthritis. J Rheumatol.

[6] Gualano B, Bonfa E, Pereira RMR, Silva CA (2017). Physical activity for paediatric rheumatic diseases: standing up against old paradigms. Nat Rev Rheumatol.

[7] Philpott J, Houghton K, Luke A (2010). Physical activity recommendations for children with specific chronic health conditions: juvenile idiopathic arthritis, hemophilia, asthma and cystic fibrosis. Paediatr Child Health.

[8] Cavallo S, Brosseau L, Toupin-April K, Wells GA, Smith CA, Pugh AG (2017). Ottawa panel evidence-based clinical practice guidelines for structured physical activity in the management of juvenile idiopathic arthritis. Arch Phys Med Rehabil.

[9] Kuntze G, Nesbitt C, Whittaker JL, Nettel-Aguirre A, Toomey C, Esau S (2018). Exercise therapy in juvenile idiopathic arthritis: a systematic review and meta-analysis. Arch Phys Med Rehabil.

[10] Takken T, van Brussel M, Engelbert RH, van der Net JJ, Kuis W, Helders PP (2008). Exercise therapy in juvenile idiopathic arthritis: a Cochrane Review. Eur J Phys Rehabil Med.

[11] Catania H, Fortini V, Cimaz R (2017). Physical exercise and physical activity for children and adolescents with juvenile idiopathic arthritis: a literature review. Pediatr Phys Ther.

[12] Tan VPS, Macdonald HM, Kim S, Nettlefold L, Gabel L, Ashe MC (2014). Influence of physical activity on bone strength in children and adolescents: a systematic review and narrative synthesis. J Bone Miner Res.

[13] Lien G, Selvaag AM, Flato B, Haugen M, Vinje O, Sorskaar D (2005). A two-year prospective controlled study of bone mass and bone turnover in children with early juvenile idiopathic arthritis. Arthritis Rheum.

[14] Burnham JM, Leonard MB (2004). Bone disease in pediatric rheumatologic disorders. Curr Rheumatol Rep.

[15] Stagi S, Cavalli L, Signorini C, Bertini F, Cerinic MM, Brandi ML (2014). Bone mass and quality in patients with juvenile idiopathic arthritis: longitudinal evaluation of bone-mass determinants by using dual-energy x-ray absorptiometry, peripheral quantitative computed tomography, and quantitative ultrasonography. Arthritis Res Ther.

[16] Burnham JM, Shults J, Dubner SE, Sembhi H, Zemel BS, Leonard MB (2008). Bone density, structure, and strength in juvenile idiopathic arthritis: importance of disease severity and muscle deficits. Arthritis Rheum.

[17] Roth J, Palm C, Scheunemann I, Ranke MB, Schweizer R, Dannecker GE (2004). Musculoskeletal abnormalities of the forearm in patients with juvenile idiopathic arthritis relate mainly to bone geometry. Arthritis Rheum.

[18] Burnham JM, Shults J, Weinstein R, Lewis JD, Leonard MB (2006). Childhood onset arthritis is associated with an increased risk of fracture: a population based study using the general practice research database. Ann Rheum Dis.

[19] Sandstedt E, Fasth A, Fors H, Beckung E (2012). Bone health in children and adolescents with juvenile idiopathic arthritis and the influence of short-term physical exercise. Pediatr Phys Ther.

[20] Heaney RP (1994). The bone-remodeling transient: implications for the interpretation of clinical studies of bone mass change. J Bone Miner Res.

[21] Petty RE, Southwood TR, Manners P, Baum J, Glass DN, Goldenberg J (2004). International league of associations for rheumatology classification of juvenile idiopathic arthritis: second revision, Edmonton, 2001. J Rheumatol.

[22] Moore SA, McKay HA, Macdonald H, Nettlefold L, Baxter-Jones ADG, Cameron N (2015). Enhancing a somatic maturity prediction model. Med Sci Sports Exerc.

[23] Singh G, Athreya BH, Fries JF, Goldsmith DP (1994). Measurement of health status in children with juvenile rheumatoid arthritis. Arthritis Rheumatol.

[24] Crocker PR, Bailey DA, Faulkner RA, Kowalski KC, McGrath R (1997). Measuring general levels of physical activity: preliminary evidence for the physical activity questionnaire for older children. Med Sci Sports Exerc.

[25] Kowalski KC, Crocker P, Faulkner RA (1997). Validation of the physical activity questionnaire for older children. Pediatr Exerc Sci.

[26] Varni JW, Burwinkle TM, Szer IS (2004). The PedsQL multidimensional fatigue scale in pediatric rheumatology: reliability and validity. J Rheumatol.

[27] Duffy CM, Arsenault L, Duffy KN, Paquin JD, Strawczynski H (1997). The juvenile arthritis quality of life questionnaire--development of a new responsive index for juvenile rheumatoid arthritis and juvenile spondyloarthritides. J Rheumatol.

[28] Whitehead JR (1995). A study of children’s physical self-perceptions using an adapted physical self-perception profile questionnaire. Pediatr Exerc Sci.

[29] Barr SI (1994). Associations of social and demographic variables with calcium intakes of high school students. J Am Diet Assoc.

[30] Tanner JM. Foetus into man. Cambridge: Harvard University Press; 1978.

[31] Gabel L, Nettlefold LA, Macdonald HM, McKay HA (2018). Sex-, ethnic- and age-specific centile curves for pQCT- and HR-pQCT-derived measures of bone structure and strength in adolescents and young adults. J Bone Miner Res.

[32] Baxter-Jones ADG, Burrows M, Bachrach LK, Lloyd T, Petit M, Macdonald H (2010). International longitudinal pediatric reference standards for bone mineral content. Bone.

[33] McKay HA, MacLean L, Petit M, MacKelvie-O'Brien K, Janssen P, Beck T (2005). “Bounce at the bell”: a novel program of short bouts of exercise improves proximal femur bone mass in early pubertal children. Br J Sports Med.

[34] Macdonald HM, Kontulainen SA, Khan KM, McKay HA (2007). Is a school-based physical activity intervention effective for increasing tibial bone strength in boys and girls?. J Bone Miner Res.

[35] McKay H, Tsang G, Heinonen A, MacKelvie K, Sanderson D, Khan KM (2005). Ground reaction forces associated with an effective elementary school based jumping intervention. Br J Sports Med.

[36] Sims-Gould J, Race DL, Macdonald H, Houghton KM, Duffy CM, Tucker LB, McKay HA (2018). “I just want to get better”: experiences of children and youth with juvenile idiopathic arthritis in a home-based exercise intervention. Pediatr Rheumatol Online J.

[37] de Onis M, Onyango AW, Borghi E, Siyam A, Nishida C, Siekmann J (2007). Development of a WHO growth reference for school-aged children and adolescents. Bull World Health Organ.

[38] Sandstedt E, Fasth A, Eek MN, Beckung E (2013). Muscle strength, physical fitness and well-being in children and adolescents with juvenile idiopathic arthritis and the effect of an exercise programme: a randomized controlled trial. Pediatr Rheumatol Online J.

[39] Feldman DE, De Civita M, Dobkin PL, Malleson PN, Meshefedjian G, Duffy CM (2007). Effects of adherence to treatment on short-term outcomes in children with juvenile idiopathic arthritis. Arthritis Rheum.

[40] Singh-Grewal D, Schneiderman-Walker J, Wright V, Bar-Or O, Beyene J, Selvadurai H (2007). The effects of vigorous exercise training on physical function in children with arthritis: a randomized, controlled, single-blinded trial. Arthritis Rheum.

[41] Rapoff MA (1989). Compliance with treatment regimens for pediatric rheumatic diseases. Arthritis Care Res.

[42] Coda A, Sculley D, Santos D, Girones X, Brosseau L, Smith DR (2017). Harnessing interactive technologies to improve health outcomes in juvenile idiopathic arthritis. Pediatr Rheumatol Online J.

[43] Lelieveld OTHM, Armbrust W, Geertzen JHB, de Graaf I, van Leeuwen MA, Sauer PJJ (2010). Promoting physical activity in children with juvenile idiopathic arthritis through an internet-based program: results of a pilot randomized controlled trial. Arthritis Care Res.

[44] Armbrust W, Bos JJFJ, Cappon J, van Rossum MAJJ, Sauer PJJ, Wulffraat N (2015). Design and acceptance of Rheumates@work, a combined internet-based and in person instruction model, an interactive, educational, and cognitive behavioral program for children with juvenile idiopathic arthritis. Pediatr Rheumatol Online J.

[45] Armbrust W, Bos GJFJ, Wulffraat NM, van Brussel M, Cappon J, Dijkstra PU (2017). Internet program for physical activity and exercise capacity in children with juvenile idiopathic arthritis: a multicenter randomized controlled trial. Arthritis Care Res.

[46] Armbrust W, Lelieveld OHTM, Tuinstra J, Wulffraat NM, Bos GJFJ, Cappon J (2016). Fatigue in patients with juvenile idiopathic arthritis: relationship to perceived health, physical health, self-efficacy, and participation. Pediatr Rheumatol Online J.

[47] MacKelvie KJ, Petit MA, Khan KM, Beck TJ, McKay HA (2004). Bone mass and structure are enhanced following a 2-year randomized controlled trial of exercise in prepubertal boys. Bone.

[48] Fricke O, Weidler J, Tutlewski B, Schoenau E (2006). Mechanography--a new device for the assessment of muscle function in pediatrics. Pediatr Res.

[49] Institute of Medicine (2010). Dietary Reference Intakes for Calcium and Vitamin D.

