# Supplementary figures and images for: Feasibility and safety of a 6-month exercise program to increase bone and muscle strength in children with juvenile idiopathic arthritis
Source: Pediatr Rheumatol Online J. 2018 Oct 22;16:67. doi: 10.1186/s12969-018-0283-4 (PMC6198360; doi:10.1186/s12969-018-0283-4)

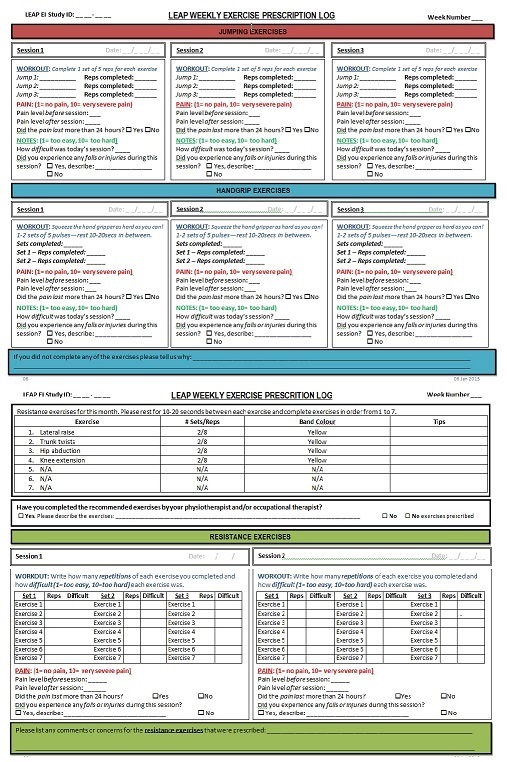

Supplement: Supplementary file 1 — Figure S1. Exercise Log. (JPG 189 kb) [file 12969_2018_283_MOESM1_ESM.jpg]
