# Supplementary material for: Feasibility and safety of a 6-month exercise program to increase bone and muscle strength in children with juvenile idiopathic arthritis
Source: Pediatr Rheumatol Online J. 2018 Oct 22;16:67. doi: 10.1186/s12969-018-0283-4 (PMC6198360; doi:10.1186/s12969-018-0283-4)
Supplement: Supplementary file 2 — Table S1. Comparison of descriptive and clinical outcomes, lumbar spine bone mineral content (BMC) z-score and total bone mineral density (Tt.BMD) at the distal tibia between those participants that withdrew and those that completed all assessments. Values are mean (standard deviation) unless otherwise indicated. Table S2. Clinical outcomes at baseline, 3-, 6- and 12-months for each participant who completed the study (n=13). (DOCX 34 kb) [file 12969_2018_283_MOESM2_ESM.docx]

**Table S1.** Comparison of descriptive and clinical outcomes, lumbar spine bone mineral content (BMC) z-score and total bone mineral density (Tt.BMD) at the distal tibia between those participants that withdrew and those that completed all assessments. Values are mean (standard deviation) unless otherwise indicated.

| **Variable** | **Completed the study (n=13)** | **Withdrew**  **(n=10)** |
| --- | --- | --- |
| Sex (# Girls/Boys) | 7/6 | 7/3 |
| Age (years) | 13.0 (2.0) | 12.1 (2.3) |
| JIA Subtype | 2 poly RF negative, 2 oligo extended, 3 oligo persistent, 3 ERA, 2 psoriatic, 2 undifferentiated | 5 ERA, 2 poly RF positive, 2 poly RF negative, 1 oligo persistent |
| Ethnicity (# Asian/Caucasian/Other) | 3/8/2 | 3/3/2 |
| Maturity offset (years) | 0.07 (1.81) | -0.10 (2.1) |
| Height (cm) | 149.7 (8.3) | 151.7 (10.1) |
| Weight (kg) | 42.6 (7.0) | 45.6 (12.6) |
| BMI (kg/m^2^) | 19.0 (2.6) | 19.5 (3.5) |
| LS BMC Z-score | -0.09 (0.64) | -0.31 (1.51) |
| Tibia Tt.BMD (mg HA/cm^3^) | 223.1 (34.1) | 222.0 (46.8) |

BMI=body mass index, LS BMC=lumbar spine bone mineral content Z-score, Tt.BMD=total bone mineral density

**Table S2.** Clinical outcomes at baseline, 3-, 6- and 12-months for each participant who completed the study (n=13)

| **ID** | **Clinical measure** | **Baseline** | **3-months** | **6-months** | **12-months** |
| --- | --- | --- | --- | --- | --- |
| 1 | Active joint count  Active enthesitis count  CHAQ (range 0-3)  Pain VAS (range 0-100)  QOL (range 0-100)  Health related QOL (range 0-100)  PAQ (range 0-5)  PedsQL Multidimensional Fatigue Scale (range 0-100)  CYPSPP - General physical self-worth (range 0-4)  Medications | 0  0  0  0  99  63  2.16  52.8  2.5  IFX MTX | 0  0  0  4  93  21  2.20  80.6  2.5  IFX MTX | 0  0  0  2  70  46  2.37  80.6  2.3  IFX MTX | 0  0  0  8  100  75  2.06  80.6  3.0  IFX MTX |
| 3 | Active joint count  Active enthesitis count  CHAQ (range 0-3)  Pain VAS (range 0-100)  QOL (range 0-100)  Health related QOL (range 0-100)  PAQ (range 0-5)  PedsQL Multidimensional Fatigue Scale (range 0-100)  CYPSPP - General physical self-worth (range 0-4)  Medications | 0  4  0.25  46  63  50  2.78  48.6  2.8  NSAID SSZ | 0  0  0  12  *  *  3.27  86.1  3.5  NSAIDSSZ | 0  0  0.25  13  96  97  2.66  62.5  3.3  NSAIDSSZ | 0  0  0.25  16  97  60  3.09  50  3.8  NSAID SSZ |
| 5 | Active joint count  Active enthesitis count  CHAQ (range 0-3)  Pain VAS (range 0-100)  QOL (range 0-100)  Health related QOL (range 0-100)  PAQ (range 0-5)  PedsQL Multidimensional Fatigue Scale (range 0-100)  CYPSPP - General physical self-worth (range 0-4)  Medications **switched for uveitis control | 0  0  0  22  50  50  3.16  61.1  3.0  IFX LEF | 0  0  0  10  80  80  3.27  77.8  3.16  ADA** LEF | 0  0  0  17  97  97  3.06  86.1  3.0  ADA LEF | 0  0  0  30  40  60  3.83  90.3  2.2  ADA LEF |
| 8 | Active joint count  Active enthesitis count  CHAQ (range 0-3)  Pain VAS (range 0-100)  QOL (range 0-100)  Health related QOL (range 0-100)  PAQ (range 0-5)  PedsQL Multidimensional Fatigue Scale (range 0-100)  CYPSPP - General physical self-worth (range 0-4)  Medications | 0  0  0.75  75  75  45  2.19  52.8  4.0  NSAID MTX | 0  0  0.125  10  90  100  3.14  80.6  4.0  NSAID MTX | 0  0  1.125  80  90  73  1.34  70.8  4.0  NSAID MTX | 1  0  1.25  79  51  22  1.63  58.3  4.0  NSAID MTX ETN PRED |
| 9 | Active joint count  Active enthesitis count  CHAQ (range 0-3)  Pain VAS (range 0-100)  QOL (range 0-100)  Health related QOL (range 0-100)  PAQ (range 0-5)  PedsQL Multidimensional Fatigue Scale (range 0-100)  CYPSPP - General physical self-worth (range 0-4)  Medications | 0  4  0.5  35  70  40  2.23  62.5  3.8  MTX | 0  3  *  *  *  *  2.17  *  *  NSAID MTX | 2  0  0.875  27  70  66  2.29  51.4  *  NSAID MTX | 0  2  0.375  12  *  *  2.34  59.7  *  NSAID MTX |
| 10 | Active joint count  Active enthesitis count  CHAQ (range 0-3)  Pain VAS (range 0-100)  QOL (range 0-100)  Health related QOL (range 0-100)  PAQ (range 0-5)  PedsQL Multidimensional Fatigue Scale (range 0-100)  CYPSPP - General physical self-worth (range 0-4)  Medications | 0  0  0  0  100  100  2.17  61.1  3.5  None | 0  0  0.375  0  100  100  3.91  47.2  2.6  None | 0  0  0.125  0  100  100  2.68  87.5  3.2  None | 0  0  0  0  100  100  1.36  76.4  2.5  None |
| 11 | Active joint count  Active enthesitis count  CHAQ (range 0-3)  Pain VAS (range 0-100)  QOL (range 0-100)  Health related QOL (range 0-100)  PAQ (range 0-5)  PedsQL Multidimensional Fatigue Scale (range 0-100)  CYPSPP - General physical self-worth (range 0-4)  Medications  *switched for uveitis treatment | 0  0  0  0  90  100  3.78  97.2  3.8  IFX LEF | 0  0  0  0  100  100  3.18  87.5  3.8  IFX LEF | 0  0  0  2  99  100  1.90  100  3.8  ADA* LEF | 0  0  0  0  100  100  3.94  86.1  4.0  ADA LEF |
| 14 | Active joint count  Active enthesitis count  CHAQ (range 0-3)  Pain VAS (range 0-100)  QOL (range 0-100)  Health related QOL (range 0-100)  PAQ (range 0-5)  PedsQL Multidimensional Fatigue Scale (range 0-100)  CYPSPP - General physical self-worth (range 0-4)  Medications | 0  0  0  0  100  100  1.93  81.9  3.5  None | 0  0  0  0  100  100  2.14  79.2  4.0  None | 0  0  0  0  83  100  2.23  70.8  3.2  None | 0  1  0  0  100  100  3.02  84.7  3.8  None |
| 16 | Active joint count  Active enthesitis count  CHAQ (range 0-3)  Pain VAS (range 0-100)  QOL (range 0-100)  Health related QOL (range 0-100)  PAQ (range 0-5)  PedsQL Multidimensional Fatigue Scale (range 0-100)  CYPSPP - General physical self-worth (range 0-4)  Medications | 0  0  0  30  80  50  2.41  44.4  3.3  None | 0  0  0.625  0  57  29  2.02  52.8  2.8  None | 0  0  0.125  1  80  50  2.59  52.8  3.7  None | 0  4  0  14  71  49  1.79  59.7  3.5  NSAID |
| 17 | Active joint count  Active enthesitis count  CHAQ (range 0-3)  Pain VAS (range 0-100)  QOL (range 0-100)  Health related QOL (range 0-100)  PAQ (range 0-5)  PedsQL Multidimensional Fatigue Scale (range 0-100)  CYPSPP - General physical self-worth (range 0-4)  Medications | 0  3  1.25  66  100  35  1.79  43.1  3.2  NSAIDMTX | 0  0  1.5  16  75  46  2.37  43.1  3.0  NSAIDMTX | 0  0  1.625  51  76  49  2.21  50.0  3.6  NSAIDMTX | 0  0  1.375  *  71  38  1.56  44.4  3.7  NSAIDMTX |
| 18 | Active joint count  Active enthesitis count  CHAQ (range 0-3)  Pain VAS (range 0-100)  QOL (range 0-100)  Health related QOL (range 0-100)  PAQ (range 0-5)  PedsQL Multidimensional Fatigue Scale (range 0-100)  CYPSPP - General physical self-worth (range 0-4)  Medications | 0  2  0.125  50  100  70  2.74  47.2  3.8  NSAIDMTX | 0  3  0.25  68  100  70  2.72  52.8  3.5  NSAIDMTX | 0  0  0.5  60  100  70  2.71  54.2  3.7  NSAIDMTX | 0  0  0  32  100  48  2.53  58.3  3.7  NSAIDMTX |
| 19 | Active joint count  Active enthesitis count  CHAQ (range 0-3)  Pain VAS (range 0-100)  QOL (range 0-100)  Health related QOL (range 0-100)  PAQ (range 0-5)  PedsQL Multidimensional Fatigue Scale (range 0-100)  CYPSPP - General physical self-worth (range 0-4)  Medications | 1  0  0.875  25  65  80  1.48  29.2  1.7  SSZ | 0  0  1.25  40  50  65  1.15  38.9  2.0  NSAID SSZ | 0  0  1.0  7  51  73  1.08  36.1  1.7  NSAID SSZ | 0  0  0.625  25  50  70  1.32  36.1  *  NSAID SSZ |
| 20 | Active joint count  Active enthesitis count  CHAQ (range 0-3)  Pain VAS (range 0-100)  QOL (range 0-100)  Health related QOL (range 0-100)  PAQ (range 0-5)  PedsQL Multidimensional Fatigue Scale (range 0-100)  CYPSPP - General physical self-worth (range 0-4)  Medications | 0  0  0  0  75  77  1.58  48.6  3.6  NSAID ETN | 7  0  *  *  *  *  2.00  *  *  ETN | 3  0  0.375  70  80  80  1.87  63.9  3.7  ETN | 2  0  0.375  0  76  80  1.39  55.6  3.3  ETN |

*Missing value. NSAID –non-steroidal anti-inflammatory medication including Naproxen, Ibuprofen. MTX – methotrexate. LEF – Leflunomide. SSZ – Sulfasalazine. ETN – Etanercept. ADA – Adalimumab. IFX – Infliximab. PRED –Prednisone.
